# The therapeutic effect of exosomes in type 2 diabetes mellitus and its complications

**DOI:** 10.3389/fmed.2025.1735392

**Published:** 2025-12-19

**Authors:** Yingying Liu, Jinpeng Wang, Yunwei Sun, Yan Chen

**Affiliations:** Department of Endocrinology, The Second Hospital of Jilin University, Changchun, China

**Keywords:** biomarkers, complications, exosomes, therapeutic effect, type 2 diabetes mellitus

## Abstract

Exosomes are a type of nanoscale extracellular vesicle secreted by cells, with a diameter of approximately 30–150 nm, which carry important biological molecules such as proteins, lipids, and RNA, functioning as messengers between cells and playing a central role in cell communication. Due to their involvement in various physiological and pathological processes and their low immunogenicity and good tissue penetration, exosomes have become a research hotspot for disease diagnostic markers and drug delivery carriers. Type 2 diabetes mellitus (T2DM) is a common chronic metabolic disorder characterized primarily by high blood sugar, whose core pathogenesis includes insulin resistance and the subsequent functional deficiency of pancreatic β-cells, which can lead to various serious complications over the long term. The review systematically elaborates on the key roles of exosomes from different cell sources in regulating T2DM and its complications, focusing on how these exosomes, through their specific microRNAs (miRNAs), proteins, and other active substances they carry, act on different key targets and thereby regulate core pathological processes such as insulin signaling pathways, inflammatory responses, cell apoptosis, fibrosis, and angiogenesis. Through the review of existing evidence, we aim to reveal the complex network of exosomes as intercellular messengers and provide a solid theoretical basis for their development as new diagnostic markers and targeted therapeutic strategies.

## 1 Introduction

Type 2 diabetes mellitus (T2DM) is a group of metabolic diseases characterized by chronic hyperglycemia, whose core mechanisms involve insufficient insulin secretion and insulin resistance. The typical clinical manifestations are “three more and one less,” namely, increased thirst, increased appetite, increased urination, and weight loss. Currently, T2DM has become a major global public health issue with a rapidly growing prevalence rate, closely related to obesity, unhealthy lifestyles, and population aging (1). If blood sugar levels remain poorly controlled over a long period, it can lead to severe chronic complications in multiple systems and organs throughout the body. These mainly include microvascular complications, including diabetic retinopathy (DR) and diabetic neuropathy (DN), as well as macrovascular complications, including atherosclerosis, seriously endangering the quality of life and lifespan of patients (2).

In recent years, exosomes, a type of nano-sized extracellular vesicle secreted actively by cells, have attracted widespread attention in the field of metabolic disease research as a key medium for intercellular information transmission (3). These microvesicles, with diameters of approximately 30–150 nm, form a complex intercellular communication network in the body by carrying biologically active molecules such as proteins, lipids, and nucleic acids (4), thereby participating in the regulation of various physiological and pathological processes (5). The biogenesis process of exosomes is a highly coordinated intracellular event. It begins with the endosomal pathway, where the endosomal membrane buds to form multivesicular bodies (MVBs) and eventually fuses with the plasma membrane, releasing its contents into the extracellular space. This process is precisely regulated by various molecules, such as endosomal sorting complex required for transport (ESCRT) complexes, transmembrane proteins including CD9, CD63, and CD81, and Rab GTPases (6). The molecular composition of exosomes depends on the type of parent cell and its physiological and pathological state (7), enabling them not only to reflect the condition of the source cell but also to regulate the physiological functions of the receptor cell by transferring functional molecules (8). This characteristic makes exosomes an ideal source of biomarkers and a promising therapeutic tool. Exosomes show great potential as diagnostic biomarkers. Due to their stable presence in various body fluids and their contents being able to reflect changes in metabolic status dynamically, they make them ideal choices for non-invasive diagnostic tools (9). Exosomes, due to their inherent low immunogenicity, good biocompatibility, and excellent ability to penetrate biological barriers, are regarded as highly promising drug delivery systems (10). They can be used as natural therapeutic agents or engineered to become carriers of therapeutic molecules.

This review aims to systematically present the latest progress on exosomes in the research of T2DM, focusing on their dual potential as biomarkers and therapeutic agents. Through in-depth analysis of the core role of exosomes in various complications of T2DM, exploration of current challenges, and outlook for future development, we hope to provide valuable perspectives for understanding this rapidly evolving field and promote the accelerated translation of basic research on exosomes into clinical applications.

## 2 Biological characteristics of exosomes

**Figure 1:**
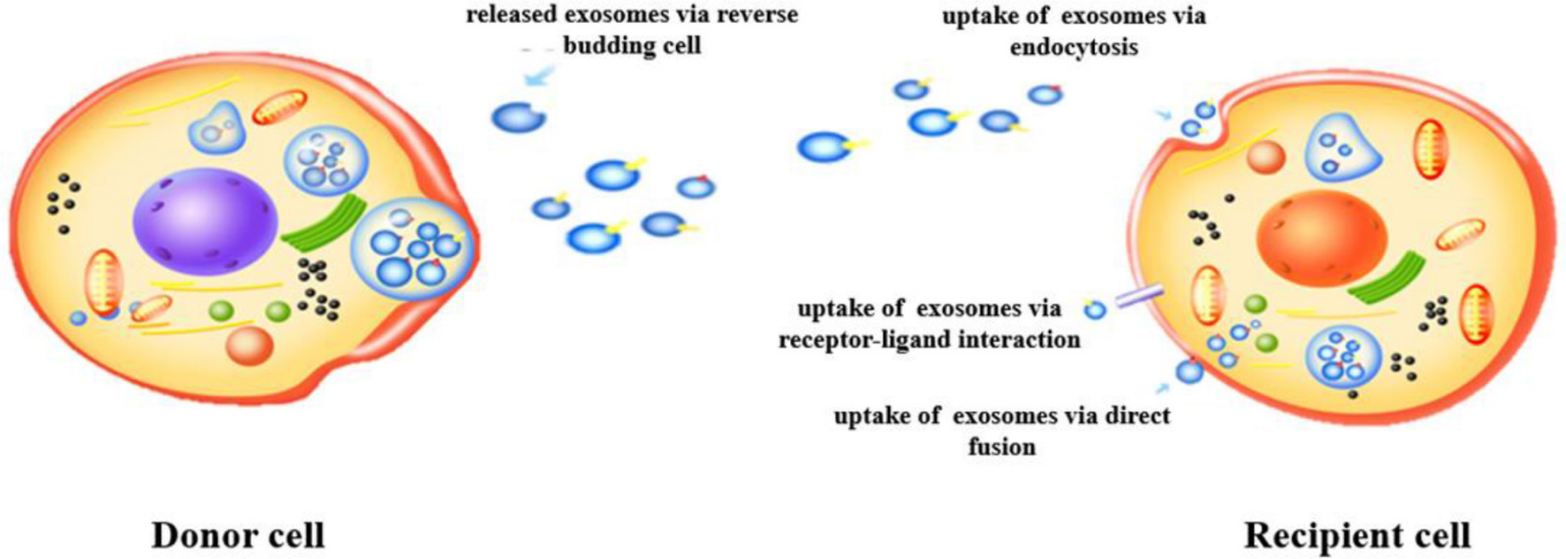
Formation and secretion of exosomes.

Exosomes are small extracellular vesicles, typically ranging from 30 to 150 nm in diameter, released by cells via an endocytic mechanism (11). Their formation begins with endocytosis, leading to the development of endosomes, which subsequently biosynthesize MVBs (12). This process involves the invagination of a portion of the cell membrane, forming an endocytic vesicle that encapsulates cytoplasmic contents, as illustrated in Figure 1. The vesicle then fuses with the endosome, and as the endosome matures, some of it transforms into MVBs. Under specific conditions, MVBs release their vesicles into the extracellular space as exosomes (13). The release of exosomes is regulated by intracellular molecular signals and external factors, such as cellular stress, metabolic status, and intercellular interactions, which control both the frequency and volume of exosome secretion (14). Additionally, the composition of exosomes is influenced by the physiological and pathological state of the parent cell, making them potential biomarkers for early disease detection and therapeutic monitoring (15).

**Figure 2:**
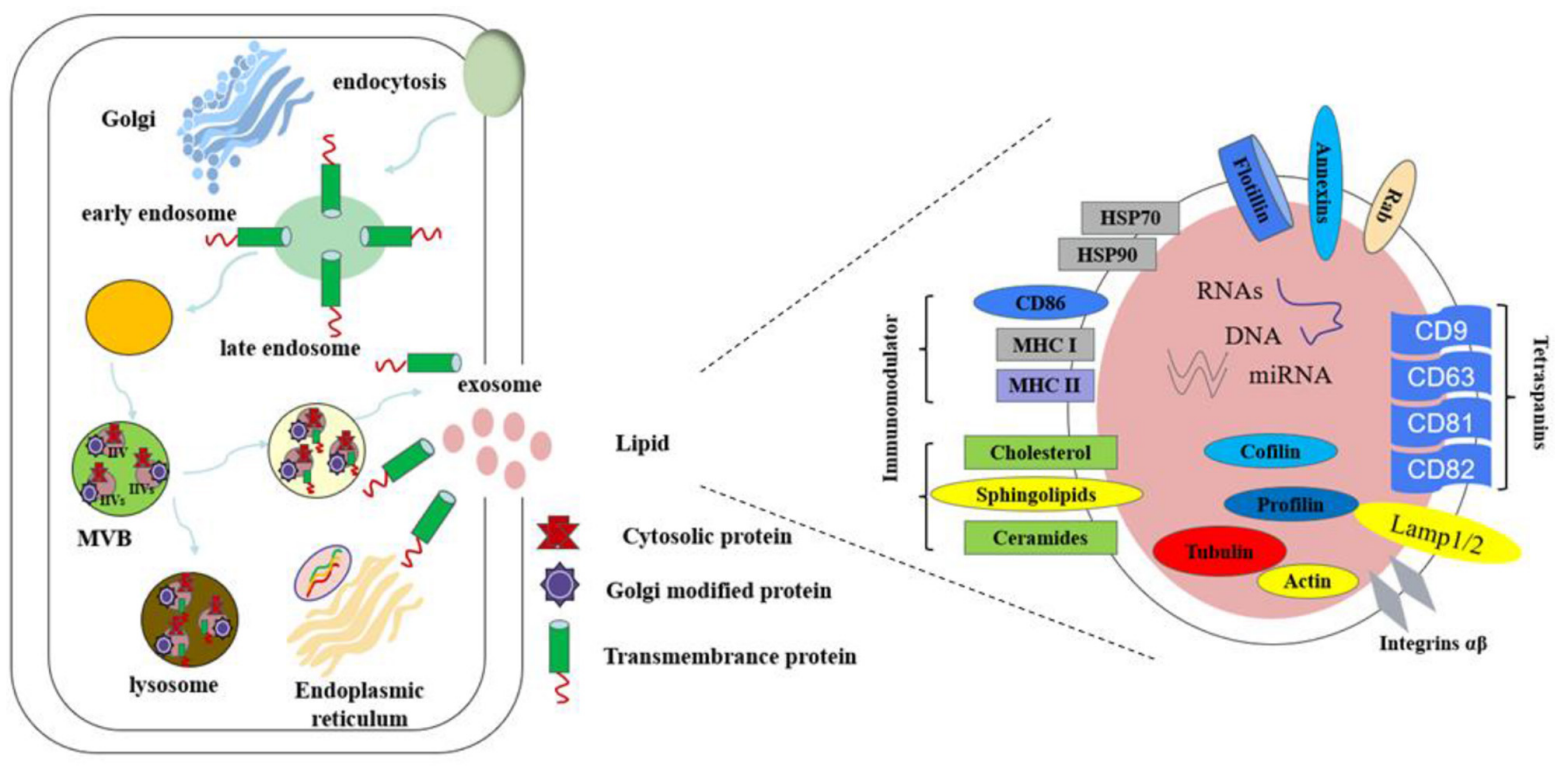
The composition of exosomes.

Exosomes contain a variety of biomolecules, including proteins, lipids, RNA (miRNA, mRNA, and lncRNA), and small amounts of DNA (16), as shown in Figure 2. These components reflect the physiological state of the parent cell and facilitate intercellular communication by transferring biological information to recipient cells, thereby influencing their functions (17). Exosomal proteins are abundant and include small GTPases, membrane transporters, kinases, and cell cycle regulators (18). Specific proteins such as CD9, CD63, and CD81 are widely recognized as exosomal markers, aiding in their isolation and purification in experimental studies (19).

The exosomal membrane consists primarily of a phospholipid bilayer enriched with lipids like cholesterol and sphingolipids. Lipid composition is crucial for the formation, structural integrity, and functional properties of exosomes. Research indicates that variations in lipid content can influence exosome distribution and intercellular interactions, modulating downstream signaling pathways (20). RNA molecules, particularly miRNA, have garnered significant research interest due to their role in gene regulation. miRNAs are small RNA molecules that regulate gene expression and participate in key cellular processes such as growth, differentiation, and apoptosis (21). The ability of exosomes to carry miRNAs makes them vital in intercellular gene regulation. By transferring specific miRNAs or mRNAs, exosomes enable efficient information exchange. For example, studies have shown that the miRNA profile in exosomes from obese individuals differs markedly from that of healthy individuals, suggesting a role for exosomes in regulating glucose and lipid metabolism (22).

Although the DNA component of exosomes is relatively minor, recent studies suggest that exosomes can carry specific genomic DNA fragments that influence immune cell states and, consequently, affect systemic metabolic balance. These DNA fragments may facilitate the transfer of genetic material between cells, further enhancing the functional significance of exosomes (23). The diverse molecular cargo within exosomes highlights their potential applications in cell communication, disease signaling, and therapeutic development. Future investigations into exosome components will provide deeper insights into their mechanisms, particularly in the context of T2DM.

**Figure 3:**
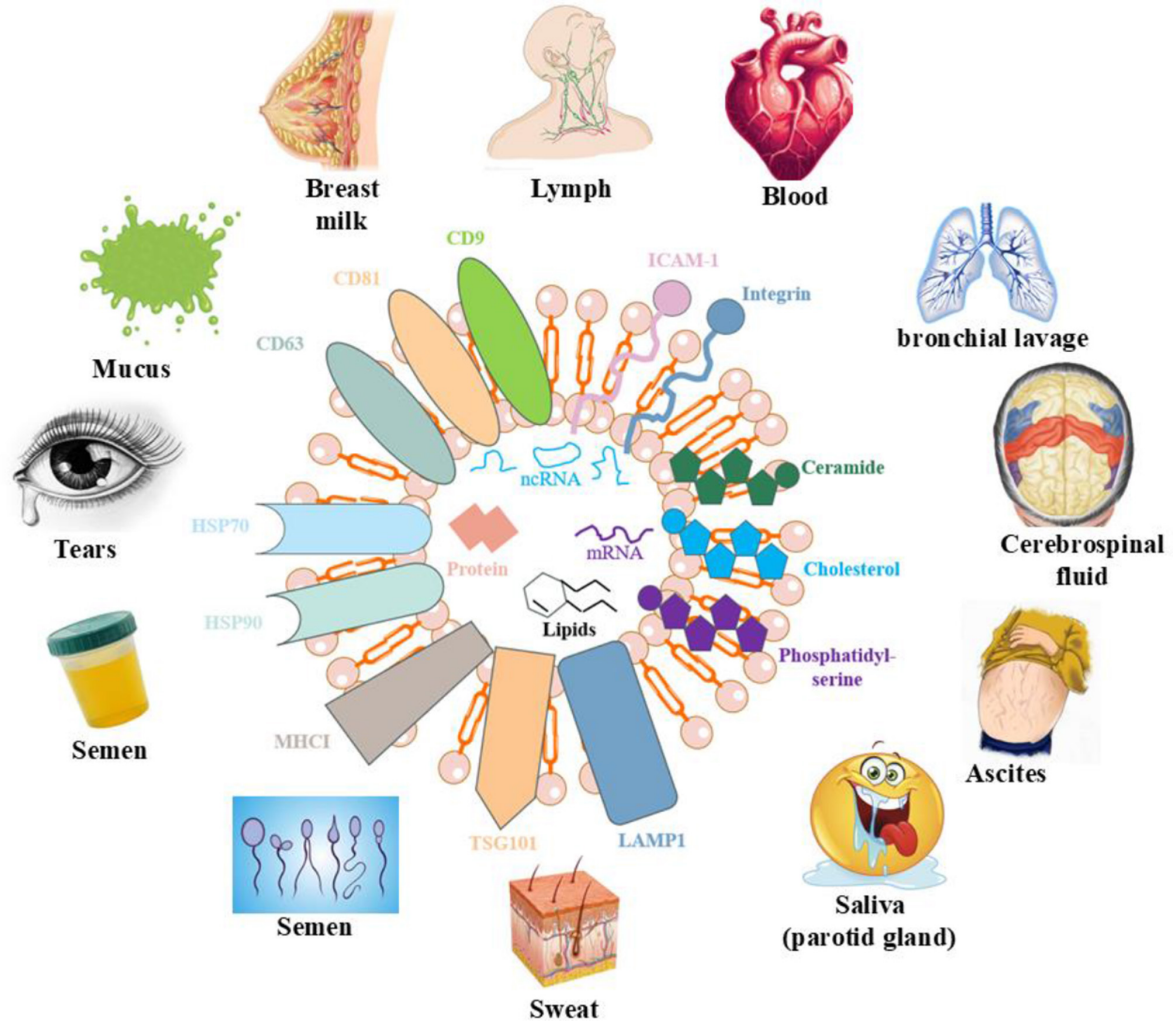
Effects of exosomes on various organs of the human body.

Exosomes not only transmit biological information but also modulate various cellular processes, as illustrated in Figure 3. They can activate signaling pathways in recipient cells, initiating diverse biological responses (24). For example, exosomes contribute to the regulation of glucose and lipid metabolism by mediating interactions among liver cells, muscle cells, and adipocytes. Furthermore, exosomes play a pivotal role in inflammatory responses, especially in chronic inflammation (25). In T2DM, high levels of free fatty acids and inflammatory mediators in obese individuals stimulate exosome production in adipose tissue, which in turn affects other cell types such as hepatocytes and myocytes, exacerbating insulin resistance (26). Understanding the mechanisms underlying exosome function under these conditions may lead to innovative therapeutic approaches.

## 3 The role of exosomes in T2DM and its complications in different physiological systems

### 3.1 Skin healing

The hyperglycemic environment in T2DM directly impairs the proliferation and migration abilities of fibroblasts and keratinocytes through mechanisms such as the polyol pathway, accumulation of advanced glycation end products (AGEs), and mitochondrial oxidative stress. At the same time, the continuous activation of inflammatory signaling pathways, such as nuclear factor kappa-B (NF-κB), leads to chronic and excessive inflammation and inhibits the expression of vascular endothelial growth factor (VEGF), thereby hindering the formation of new blood vessels. These functional dysfunctions collectively result in delayed re-epithelialization and formation of granulation tissue (27).

Multiple studies have confirmed that the exosomes secreted by mesenchymal stem cells (MSCs) extracted from various tissues, such as umbilical cord, placenta, and bone marrow, can significantly promote the healing of diabetic wounds through multiple mechanisms. Exosomes derived from MSCs can promote the upregulation of vascular endothelial growth factor receptor (VEGFR) and angiogenesis through miRNA-21-5p/miR-221-3p and a kinase transforming (AKT)/mitogen-activated protein kinase (MAPK) signaling pathways (28, 29) and also upregulate the expression of endothelial cells (ECs) tubular formation and glucose transporter by influencing the energy metabolism of vascular ECs, including cell survival ability, metabolic activity, oxidative stress, and antioxidant capacity, thereby facilitating vascular formation and glucose metabolism (30). In addition, exosomes derived from human umbilical cord mesenchymal stem cells (hUC-MSCs) can also accelerate the healing of diabetic wounds by alleviating oxidative stress damage to ECs, enhancing the regeneration of granulation tissue, and promoting angiogenesis through up-regulating the expression of VEGF and transforming growth factor β-1 (TGFβ-1) (31, 32). Liang et al. reported that exosomes derived from UCMSCs can also promote EC proliferation, migration, and angiogenesis through the miR-20b-5p/Nrf2/VEGFA axis, thereby promoting angiogenesis in diabetic ulcer wounds (33). Another study shows that the inhibitory agents derived from human placental mesenchymal stem cells (hPMSCs) from patients with gestational diabetes mellitus (GDM) can promote the proliferation and angiogenesis of ECs by targeting intercellular cell adhesion molecule-1 (ICAM-1) through miRNA-130b-3p and increase the expression of angiogenesis-related factors (34). To enhance the sustained release and bioavailability of this exosome, Varyani et al. encapsulated the exosomes derived from hPMSCs in a hydrogel. They found that compared with the exosome-only group, the wound contraction rate, new epidermal length, number of fibroblasts and blood vessels, collagen density, and antioxidant factors, including glutathione (GSH), superoxide dismutase (SOD), and catalase (CAT), in the treatment group were significantly increased (35).

Some studies have also shown that exosomes derived from adipose-derived stem cells (ADSCs) have a significant effect in the treatment of T2DM. In contrast, they can regulate macrophage polarization, anti-inflammatory and anti-apoptotic functions to improve glucose tolerance and insulin sensitivity while conferring immune regulatory functions to ECs by delivering circular Snhg11 (36, 37). In contrast, they can regulate miR-204-3p/ homeodomain-interacting protein kinase 2 (HIPK2) and miR-1248 to promote ECs proliferation, migration, and angiogenesis and upregulate the mRNA and protein levels of pro-angiogenic factors VEGF-A, angiopoietin-1 (Angpt-1), and TGF-β, thereby improving diabetic foot ulcers (38, 39). Song et al. (40) also incorporated it into gelMA hydrogel to form a mixture and found that gelMA-HExo could enhance the migration, proliferation, and vascular regeneration potential of vascular ECs through miR-144-3p/nuclear factor erythroid 2-related factor 2 (NFE2L2)/hypoxia-inducible factor 1-α(HIF1α).

A study by Ji et al. showed that macrophage-derived exosomes induced by AGEs could impair the proliferation, migration, and tube formation of ECs through the miR-22-5p/forkhead box protein P1 (FOXP1) signaling axis, while increasing monocyte adhesion and the release of proinflammatory cytokines. It can be seen that miR-22-5p is a potential therapeutic target for diabetic skin healing, as inhibiting it can release exosomes and fibroblast growth factors, accelerating oxidative diabetic wound healing (41). Platelet-rich plasma (PRP)-derived exosomes encapsulating hydrogels inhibit fibroblast ferroptosis by upregulating FosB expression, resulting in increased cell survival, reduced oxidative stress, and elevated iron levels. At the same time, the expressions of glutathione peroxidase 4 (GPX4) and solute carrier family 7 member 11 (SLC7A11) are upregulated, thereby accelerating wound healing (42). Since the role of ECs in skin healing is of paramount importance, many researchers are now focusing on exosomes derived from these. They reported that exosomes derived from ECs can accelerate the healing of skin wounds in diabetic mice by promoting the expression levels of miRNA-221-3p, VEGF, CD31, and Ki67, which are vascularization-related factors and cell proliferation markers. Bioinformatics analysis indicated that miRNA-221-3p may be involved in the Advanced Glycation End product–Receptor for AGE (AGE–RAGE) signaling pathway in diabetes complications, as well as the cell cycle and p53 signaling pathways (43). Furthermore, in exosomes derived from ECs, they reprogram DNA methyltransferase 1 by promoting the expression of endothelial nitric oxide synthase (eNOS), hCAT-1, VEGF, and ICAM-1, thereby reducing the expression of exosome proteins related to vascular complications, such as Thrombospondin1, Pentraxin3, and Cystatin C (44, 45). Exosomes derived from hypoxic urine-derived stem cells and fibroblast stem cells negatively regulate the HIF-1α/VEGF/VEGFR signaling pathway through miR-486-5p and miRNA-24-3p, thereby promoting the proliferation, migration, and tubal formation of ECs (46, 47).

In recent years, engineered exosomes have gained attention due to their customization. Zhai et al. discovered that engineered exosomes containing interleukin-4 (IL-4) have a significant synergistic effect in regulating immune responses and enhancing angiogenesis. In contrast, they can increase the survival rate, migration, and tubule formation ability of endothelial cells (HUVECs), and, in contrast, they can cause macrophages to polarize toward the anti-inflammatory M2 phenotype, achieving complete repair of diabetic wounds (48). Plant-derived exosomes, due to their excellent biocompatibility and stability, can serve as potential therapeutic agents. Studies have found that mango–ginger-derived plant-derived nanovesicles (MGDNVs) can significantly promote keratinocyte migration *in vitro* and *in vivo* by inducing the expression of proteins similar to follicular protein 1 (FSTL1). It can be seen that FSTL1 is a key factor promoting migration in wound healing. The study confirmed that MGDNV can become a natural and cost-effective local alternative to traditional biological agents for the treatment of chronic wounds (49).

Exosomes, as nanoscale messengers between cells, play a crucial role in regulating the healing process of diabetic skin wounds by delivering various bioactive substances, as shown in Table 1. Their inherent advantages, such as low immunogenicity, high stability, and ease of storage and transportation, make them a highly promising new therapeutic strategy.

**Table 1:** The mechanism of kinds of exosomes regarding skin healing of T2DM.

**Source of exosomes**	**Mechanism**	**Signal path or receptor**	**References**
MSCs	• Promote ischemic tissue repair and angiogenesis	AKT-MAPK/miRNA-21-5p	(28)
^*^BMSCs	• Promote the angiogenesis, cell viability, and migration	miR-221-3p	(29)
MSCs	• Promote cell viability, metabolic activity, and antioxidant capacity	–	(30)
hUC-MSCs	• Ameliorate Oxidative Stress and promote angiogenesis	–	(31)
hUC-MSCs	• Enhance regeneration of granulation tissue • Upregulate expression of VEGF and TGFβ-1	–	(32)
UC-MSCs	• Promote cell proliferation, migration, and angiogenesis	CircHIPK3/miR-20b-5p/Nrf2/VEGFA	(33)
hPMSCs	• Regulate proliferation, migration, and angiogenesis	microRNA-130b-3p/ ICAM-1	(34)
hPMSCs	• Anti-inflammation • Anti-oxidative stress	–	(35)
ADSCs	• Regulate macrophage polarization	miR-222-3p/ Bcl2l11/Bim	(36)
ADSCs	• Promote proliferation, migration, and angiogenesis	miR-204-3p/HIPK2	(38)
ADSCs	• Upregulate pro-angiogenic factors	miR-1248	(39)
ADSCs	• Enhance autophagy	miR-144-3p/NFE2L2/HIF1α	(40)
Macrophages	• Regulate proliferation, migration, and tube formation capabilities	miR-22-5p	(41)
PRP	• Inhibit fibroblast ferroptosis	FosB	(42)
HUVECs	• Promote vascular formation and cell proliferation	AGE-RAGE/miRNA-221-3p	(43)
HUVECs	• Regulate endothelial dysfunction	–	(44)
HUVECs	• Epigenetic reprogramming	–	(45)
^*^hUSCS	• Promote endothelial cell proliferation, migration, and tube formation	miR-486-5p/HIF-1α	(46)
Fibroblasts	• Improve neovascularization and healing dynamics	miRNA-24-3p/HIF-1α/VEGF/VEGFR	(47)
Engineered exos of IL-4	• Regulate immunomodulatory and pro-angiogenic efficacy	–	(48)
MGDNVs	• Promote keratinocyte migration	FSTL1	(49)
M2 macrophages	• Anti-apoptosis	miR-223	(99)
^*^OSCC	• Promote the proliferation and migration	–	(100)
^*^SHED	• Promote angiogenesis	–	(51)
Skeletal muscle	• Anti-reactive oxygen species production • Promote angiogenesis	Nrf2/NF-κB	(101)

^*^hUSCS, hypoxic urine-derived stem cells; OSCC, oral squamous cell carcinoma; BMSCs, bone marrow mesenchymal stem cells; SHED, stem cells from human exfoliated deciduous teeth.

### 3.2 Liver

Insulin resistance leads to fatty degeneration of the liver. High insulin and blood sugar levels enhance lipid synthesis and uptake in the liver, while inhibiting fatty acid oxidation, resulting in abnormal fat accumulation within liver cells and the development of non-alcoholic fatty liver disease. Additionally, chronic high blood sugar triggers oxidative stress and endoplasmic reticulum stress, driving inflammatory responses and activation of hepatic stellate cells, which jointly exacerbate liver inflammation and fibrosis (50).

Studies have shown that exosomes derived from bone marrow macrophages (BMMs) can alleviate insulin resistance in hepatocytes and enhance insulin sensitivity through miR-143-5p and miR204/Elovl6. Meanwhile, miR204-mediated interactions between adipocytes and macrophages can regulate insulin sensitivity in adipocytes (51). The exosomes secreted by ADSCs from high-sugar diet sources promote inflammation in adipocytes by inhibiting nuclear factor erythroid 2-related factor 2 (Nrf2) expression and increase the expression of NOD-like receptor thermal protein domain associated protein 3 (NLRP3) inflammasome-related proteins (52), while the exosomes secreted by aADSCs from high-fat diet sources induce the formation of non-alcoholic fatty liver disease, lipid accumulation and inflammation (53), from which can be seen that the exosomes derived from ADSCs play a significant role in regulating liver lipid metabolism. In recent years, plant-derived exosomes have been increasingly reported. Lipid-derived exosome-like nanovesicles that are derived from orange peels can alleviate liver steatosis in T2DM by regulating lipid metabolism and the intestinal microbiota (54). Natural exosomes-like nanoparticles in mung bean (MELN) sprouts can alleviate the progression of T2DM by activating the phosphatidylinositol 3-kinase (PI3K)/Akt/glucose transporter type 4 (GLUT4)/glycogen synthase kinase-3β (GSK-3β) signaling pathway, which reduces fasting blood glucose levels, triglycerides (TG), total cholesterol (TC) and reduces the inflammatory infiltration and oxidative stress levels in liver cells, thereby improving the survival ability of liver cells (55). Dual-Carriers of Tartary Buckwheat-Derived Exosome-Like Nanovesicles (TB-ELNs) can regulate glucose metabolism in the gut-liver axis and enhance the phagocytic function of the endoplasmic reticulum, thereby reducing damage to the gastrointestinal tract (56). Exosomes derived from liver and milk can, respectively, inhibit PHLPP2 and the mammalian target of rapamycin (mTOR) in adipocytes through miR-130a-3p and miR-101-3p, thereby alleviating glucose tolerance, demonstrating that they are key players in the body's energy homeostasis (57, 58). A clinical study conducted in 2022 demonstrated that by using a mass spectrometry-based method to analyze the global proteome and phosphorylated proteome of exosomes in patients with prediabetes and T2DM, the results showed that the circulating exosomes in diabetic patients contained higher levels of specific phosphorylated kinases, such as protein kinase b-alpha (AKT1), glycogen synthase kinase 3β (GSK3B), LYN, mitogen-activated protein kinase 2 (MAP2K2), myosin light chain kinase (MYLK_, and Protein Kinase C Delta (PRKCD). Moreover, the activated kinase system may be systematically distributed throughout the body, which provides new insights into the pathobiology of T2DM (59).

Exosomes, as key carriers of intercellular communication, have shown great potential in the treatment of liver diseases in T2DM, as shown in Table 2. By carrying specific miRNAs, proteins, they precisely regulate the fate of liver cells, providing novel strategies for drug delivery and liver disease intervention. Their efficacy has been confirmed in disease models, including fatty liver and liver fibrosis.

**Table 2:** The mechanism of kinds of exosomes regarding liver lesion of T2DM.

**Source of exosomes**	**Mechanism**	**Signal path or receptor**	**References**
ADSCs and macrophages	• Anti-inflammation	Nrf2/miR-500a-5p	(52)
ADSCs	• Inhibit lipid accumulation and apoptosis	AMPK α1	(53)
Tangerine peel	• Regulate lipid metabolism and intestinal microflora	–	(54)
Mung bean	• Anti-inflammation • Anti-oxidative stress	PI3K/Akt/GLUT4/GSK-3β	(55)
Dual-carriers of tartary buckwheat	• Activate the phagocytic function of the endoplasmic reticulum	–	(56)
Liver	• Improve glucose intolerance	PHLPP2 /miR-130a-3p	(57)
Milk	• Regulate proliferation	mTOR/miR-101-3p	(58)
Circulating extracellular	• Distribute activated kinases	OXPHOS	(59)
Macrophages	• Promote insulin resistance	MKP5/ miR-143-5p	(102)

### 3.3 Pancreas

The core mechanisms underlying pancreatic lesions in T2DM are the progressive β-cell failure and the formation of pancreatic amyloidosis. In the context of insulin resistance, β-cells undergo compensatory proliferation and secrete excessive insulin, eventually leading to increased endoplasmic reticulum stress and oxidative stress. Lipotoxicity and glycolipotoxicity jointly induce β-cell apoptosis, while abnormal deposition of pancreatic amylin forms pancreatic amyloid substances, further damaging the pancreatic structure (60). This vicious cycle ultimately results in a reduction in β-cell numbers and insulin secretion defects, leading to irreversible pathological changes.

Exosomes derived from MSCs, in contrast, protect β-cells from hypoxia-induced apoptosis and alleviate endoplasmic reticulum stress by regulating miR-21 and inhibiting p38 MAPK phosphorylation, significantly increasing the survival rate of β-cells (61) and, in contrast, they enhance the function and quantity of β-cells, as well as reduce random blood glucose levels, improve glucose and insulin tolerance, and increase insulin secretion, which mechanism is achieved through AKT/extracellular signal-regulated kinase (ERK) (62). Furthermore, multiple studies have shown that some miRNAs play a crucial role in protecting the pancreas. Exosomes derived from ADSCs protect β-cells function by carrying miR-138-5p and regulating the sex-determining region Y (SRY)-box transcription factor 4 (SOX4)-mediated Wnt/β-catenin pathway (63), exosomes derived from M1 macrophages inhibit β-cell insulin secretion by targeting sirtuin 2 (SIRT2) with miR-212-5p and inhibiting the Akt/GSK-3β/β-catenin pathway (64) and Pancreatic cancer-derived exosomes inhibit β-cell function and reduce insulin secretion by targeting adenylate cyclase 1 (ADCY1) and exchange protein directly activated by cAMP 2 (EPAC2) (65). Besides, exosomes derived from liver and muscle can respectively alleviate β-cell damage through AKT kinase and mTOR signaling pathways, significantly increasing their cell survival rate (66, 67).

Exosomes offer innovative therapeutic ideas for pancreatic lesions in T2DM, as shown in Table 3. These natural nanovesicles can precisely improve the function of pancreatic β-cells by delivering active components, such as miRNAs. This therapy, based on intercellular communication, can reshape the pancreatic microenvironment, opening a new therapeutic pathway to prevent the progressive failure of β-cells and reverse the progression of diabetes.

**Table 3:** The mechanism of kinds of exosomes regarding pancreas lesion of T2DM.

**Source of exosomes**	**Mechanism**	**Signal path or receptor**	**References**
MSCs	• Anti-apoptosis, • Reduce endoplasmic reticulum stress	p38 MAPK	(61)
MSCs	• Suppresses ferroptosis	AKT/ERK	(62)
ADSCs	• Anti-apoptosis • Enhance insulin sensitivity	SOX4/Wnt/β-actin/miR-138-5p	(63)
M1 macrophages	• Increase insulin secretion	Akt/GSK-3β/β-catenin	(64)
Pancreatic cancer	• Regulate β-cell dysfunction	microRNA-19a	(65)
Liver	• Affect insulin expression	AKT	(66)
Muscle	• Reduce oxidative stress • Enhance autophagy • Anti-apoptosis	Akt/mTOR	(67)
Macrophages	• Enhance insulin sensitivity	miR204/Elovl6	(103)

### 3.4 Diabetic nephropathy

T2DM primarily causes kidney damage through metabolic and hemodynamic pathways. Long-term high blood sugar leads to the accumulation of AGEs, activation of protein kinase C, and hyperactivity of the polyol pathway, all of which jointly cause damage to the glomerular filtration barrier. At the same time, high blood sugar, in conjunction with hypertension, causes high intra-glomerular pressure and hyperfiltration, continuously activating the renin-angiotensin system, promoting inflammatory responses and fibrosis processes, ultimately leading to glomerular sclerosis and tubulointerstitial fibrosis and gradually developing into diabetic nephropathy (68).

Exosomes derived from MSCs can not only induce autophagy in glomerular endothelial cells through the mammalian target of rapamycin (mTOR) signaling pathway to improve DN (69), but also inhibit pyroptosis in proximal tubular cells of the kidney through miR-30e-5p (70), which is the first time the role of exosomes in inhibiting cell pyroptosis in T2DM has been reported. Furthermore, the exosomes derived from BMSCs alleviate DN in rats by inhibiting cell apoptosis and reducing inflammatory levels. The results of the *in vivo* experiments showed a significant decrease in the levels of glucose (GLU), creatinine (Cr), and blood urea nitrogen (BUN) (71). Ning et al. reported that exosomes derived from glomerular ECs themselves can also carry miR-30a-5p and regulate angiogenesis through the Notch1/VEGF signaling pathway (72). These findings suggest that exosome miR-30-5p may become a potential strategy for treating DN. Besides MSC-derived exosomes, exosomes secreted by ADSCs can also inhibit podocyte apoptosis and slow down DR by activating autophagy flux (73, 74). Studies have confirmed that the expression of miRNA-615-3p and miRNA-3147 in urine-derived exosomes is closely related to the inflammation and fibrosis of DN and is positively correlated with serum cystatin C, plasma TGF-β1, creatinine, blood urea nitrogen (BUN), protein creatinine ratio (PCR), and 24-h urine protein, and negatively correlated with eGFR and albumin (75).

In conclusion, exosomes demonstrate multi-dimensional therapeutic potential in the intervention of DR, as shown in Table 4. They can simultaneously regulate multiple pathological processes of the renal microenvironment: not only inhibiting the release of inflammatory factors and oxidative stress damage, but also maintaining the structural integrity of podocytes and blocking the activation of renal fibrosis signaling pathways. Their natural targeting properties and low immunogenicity offer new avenues for the precise treatment of diabetic nephropathy, especially their unique advantage in delaying glomerular sclerosis and improving renal function indicators.

**Table 4:** The mechanism of kinds of exosomes regarding DN of T2DM.

**Source of exosomes**	**Mechanism**	**Signal path or receptor**	**References**
MSCs	• Regulate autophagy	mTOR	(69)
BMSCs	• Inhibit pyroptosis	miR-30e-5p/ ELAVL1	(70)
BMSCs	• Anti-apoptosis • Anti-inflammation	–	(71)
^*^GECs	• Regulate angiogenesis	Notch1/VEGF /miR-30a-5p	(72)
ADSCs	• Promote autophagy flux and inhibit apoptosis	miR-486/Smad1/mTOR	(73)
ADSCs	• Anti-apoptosis • Anti-inflammation	USP25	(74)
hUSCS	• Inhibit inflammation and fibrosis	miRNA-615-3p and miRNA-3147	(75)

^*^GECs, Glomerular endothelial cells.

### 3.5 Neurological disorders

In contrast, persistent hyperglycemia leads to activation of the polyol pathway, accumulation of advanced glycation end products, activation of the protein kinase C pathway, and oxidative stress response, all of which jointly cause metabolic disorders in neurons and Schwann cells. In contrast, high blood sugar damages microvessels, resulting in narrowing and ischemia of nerve-feeding vessels. These pathological changes ultimately lead to demyelination of peripheral nerve fibers and axonal degeneration, triggering the typical diabetic peripheral neuropathy (76).

The exosomes secreted by BMSCs can exert neuroprotective functions through multiple pathways. First, they can enhance neural conduction velocity and increase the number of epineural nerve fibers (IENFs), myelin thickness, and axon diameter in the sciatic nerve by targeting miRNAs through the Toll-like receptor 4 (TLR4)/NF-κB signaling pathway, and can also reduce and increase the markers of M1 and M2 macrophage phenotypes (77). Second, they can target and weaken the neurological dysfunction after diabetic cerebral hemorrhage through miR-129-5p, promoting neuroinflammation and functional recovery (78). Third, these exosomes can be transferred to the damaged neuronal and astrocyte regions to improve cognitive impairment caused by diabetes and restore normality (79). Additionally, a clinical study derived from adipose tissue exosomes shows that through Mendelian randomization and multicenter population studies, it was confirmed that 13 exosomal miRNAs were significantly upregulated in the adipose tissue of T2DM patients, among which the increase of miR-125a-5p was associated with Alzheimer's disease (AD), risk of amnestic mild cognitive impairment (aMCI) and reduced volume of the left hippocampus. This confirmed that the serum exosomal miR-125a-5p extracted from adipose tissue can serve as a pathogenic biomarker for cognitive impairment in T2DM patients (80).

Exosomes precisely target the core pathological links of neurological disorders regarding T2DM through multiple mechanisms, including regulating neuroinflammation, promoting synaptic remodeling, and clearing abnormal protein aggregates, as shown in Table 5. This intercellular communication carrier provides a new strategy to break through traditional treatment bottlenecks and has significant application prospects in neural regeneration and functional recovery.

**Table 5:** The mechanism of kinds of exosomes regarding neurological disorders of T2DM.

**Source of exosomes**	**Mechanism**	**Signal path or receptor**	**References**
MSCs	• Alleviate neurovascular dysfunction • Anti-suppression of proinflammatory genes	TLR4/NF-κB	(77)
BMSCs	• Anti-inflammation	miR-129-5p	(78)
BMSCs	• improve cognitive impairment	–	(79)
Serum from adipose	• Serum exosomal	miR-125a-5p	(80)
BMSCs and ^*^HUCBCs	• Angiogenesis, neurogenesis, vascular remodeling • Modulate inflammatory and immune responses	–	(104)

^*^HUCBCs, human umbilical cord blood cells.

### 3.6 Osteoporosis

In patients with T2DM, elevated blood sugar and the accumulation of AGEs directly inhibit the function of osteoblasts and reduce bone quality. At the same time, inflammatory factors released during chronic inflammation further disrupt the balance between bone formation and bone resorption. Moreover, the combined effects of insulin resistance and oxidative stress interfere with the normal metabolism and repair capabilities of the bones, resulting in reduced bone formation, increased bone fragility, and significantly delaying the bone regeneration process (81).

In terms of bone loss, exosomes derived from damaged liver tissues are significantly enriched in the periodontal region, inducing pyroptosis in periodontal ligament cells (PDLCs). Among them, fatty acid synthase (Fasn) leads to the ectopic synthesis of fatty acids in PDLCs and activates the cleavage of gasdermin D. The results show that the deletion of Fasn in the liver can effectively alleviate the pyroptosis of PDLCs and reduce bone loss. This study reveals the organ communication mediated by exosomes in the “liver-bone” axis, providing insights for the prevention and treatment of diabetes-related bone diseases in the future (26). Salivary exosomes can inhibit local inflammatory responses through miR-25-3p, reducing periodontal alveolar bone loss by approximately 34% (82). In terms of osteogenesis, Schwann cell-derived exosomes improve bone parameters around dental implants in T2DM rats through the miR-15b-5p/Txnip signaling axis (83), BMSC-derived exosomes promote bone regeneration by regulating the miR-17-5p/mothers against decapentaplegic homolog 7 (SMAD7) axis (84) and stem cell exosomes overexpressing Smpd3 promote osteogenesis and differentiation of BMSCs derived from the jawbone by activating jawbone autophagy and inhibiting macrophage polarization and oxidative stress caused by blood glucose fluctuations (85), which collectively provide insights into the molecular mechanism of peripheral nerve regulation of bone regeneration in diabetic patients.

Exosomes, as a key medium for intercellular communication, have shown crucial potential in treating diabetic osteogenic dysplasia, as shown in Table 6. In contrast, they promote the proliferation and differentiation of osteoblasts and enhance their mineralization function. In contrast, they inhibit the activity of osteoclasts. This multi-targeted mechanism can effectively improve microcirculation disorders and inflammatory states in the context of high blood sugar, providing a novel therapeutic strategy for reversing bone loss and accelerating bone defect repair.

**Table 6:** The mechanism of kinds of exosomes regarding osteoporosis of T2DM.

**Source of exosomes**	**Mechanism**	**Signal path or receptor**	**References**
Liver	• Reduce pyroptosis	–	(26)
Saliva	• Anti-inflammation	miR-25-3p	(82)
Schwann cells	• Anti-inflammation • Anti-oxidative stress	miR-15b-5p	(83)
BMSCs	• Affect bone regeneration	miR-17-5p/SMAD7	(84)
BMSCs	• Promote the osteogenesis and differentiation • Activate autophagy • Inhibit macrophage polarization • Aniti-oxidative stress	–	(85)

### 3.7 Other complications

Exosomes also have therapeutic effects on other physiological organ complications. Due to the limited research available, we will present these together. Regarding Diabetic cardiomyopathy (DCM), Lin et al. confirmed that exosomes derived from macrophages improve the formation of resistance proteins and neointimal hyperplasia through miRNA-150-5p, exerting a protective effect on the heart (86). However, Govindappa believes that macrophage exosomes significantly enhance the inflammatory and pro-fibrotic responses of mouse fibroblasts and myocardial fibrosis. Only by knocking down human antigen R (HuR) in the exosomes can the angiotensin II-induced myocardialization response be inhibited and the left ventricular function be maintained (87). A clinical study stated that they isolated serum exosomes from 147 patients with and without diabetes and found that CD14 from serum exosomes was associated with T2DM. T2DM may promote the increase of CD14 protein in exosomes, thereby increasing the susceptibility to atherosclerosis. However, the analysis of the contents of exosomes from diabetic patients is still in its early stage, so a comprehensive characterization is crucial for it to serve as a biomarker or to analyze its potential contribution to diabetic complications (88).

Exosomes also exert a promising effect against DR. Exosomes derived from MSCs carry miR-125a-5p, in contrast, they enhance the survival rate, proliferation and migration of corneal ECs. In contrast, they inhibit the endoplasmic reticulum stress induced by high glucose, thereby jointly improving diabetic keratopathy (DK) (89). Additionally, exosomes derived from adipocytes regulate the levels of apoptosis, inflammation, and oxidative stress in mouse retinal microvascular endothelial cells (mRMECs) by modulating the miR-361-5p/TRAF3 axis, making them a potential target for treating microvascular complications induced by diabetes (90). Exosomes derived from Müller cells regulate microglial cell polarization in DR by carrying lncRNAs, including miR-320-3p, miR-221-3p, and miR-574-5p, promoting the polarization of microglia to the M1 phenotype (91).

Furthermore, adipocyte exosomes carry miR-4472, which downregulates the expression level of myocyte enhancer factor 2D (MEF2D), inhibiting the glucose consumption and uptake capacity of skeletal muscle cells (92). Garlic-like exosome-like nanoparticles (GaELNs) increase the level of phosphatidylcholine through the gut–brain axis, inhibiting cGas and STING-mediated inflammation as well as the interaction between GLP-1R and the insulin pathway (93). MSCs-derived exosomes alleviate the damage of human pulmonary microvascular endothelial cells (hPMECs) induced by high glucose and lipopolysaccharide through the Nrf2/heme oxygenase 1 (HO-1) pathway, reducing the levels of inflammation and ferroptosis (94).

Therefore, exosomes derived from different cell sources also play certain therapeutic roles in the heart, lungs, retina, skeletal muscle, and intestinal microbiota, as shown in Table 7. However, current research in these areas is relatively limited. In the future, more studies can be conducted to further explore their mechanism of action.

**Table 7:** The mechanism of kinds of exosomes regarding other complications of T2DM.

**Source of exosomes**	**Mechanism**	**Signal path or receptor**	**References**
Macrophages	• Ameliorate resistin and neointimal hyperplasia formation	miRNA-150-5p	(86)
Macrophages	• Attenuate fibrosis and inflammation	–	(87)
Serum from T2DM	• Increase atherogenic index of plasma	CD14	(88)
MSCs	• Regulate endoplasmic reticulum stress	miR-125a-5p	(89)
ADSCs	• Anti-inflammation • Anti-oxidative stress	LINC00968/miR-361-5p/TRAF3	(90)
Müller Cells	• Regulate microglia polarization	miR-320-3p、miR-221-3p 和 miR-574-5p	(91)
ADSCs	• Inhibit glucose uptake	MEF2D/GLUT4/miR-4472	(92)
Garlic	• Regulate gut bacteria via the gut-brain axis	–	(93)
MSCs	• Anti-inflammation • Inhibit ferroptosis	Nrf2/HO-1	(94)
hUC-MSCs	• Anti-inflammation	INS/SOD1	(105)

**Figure 4:**
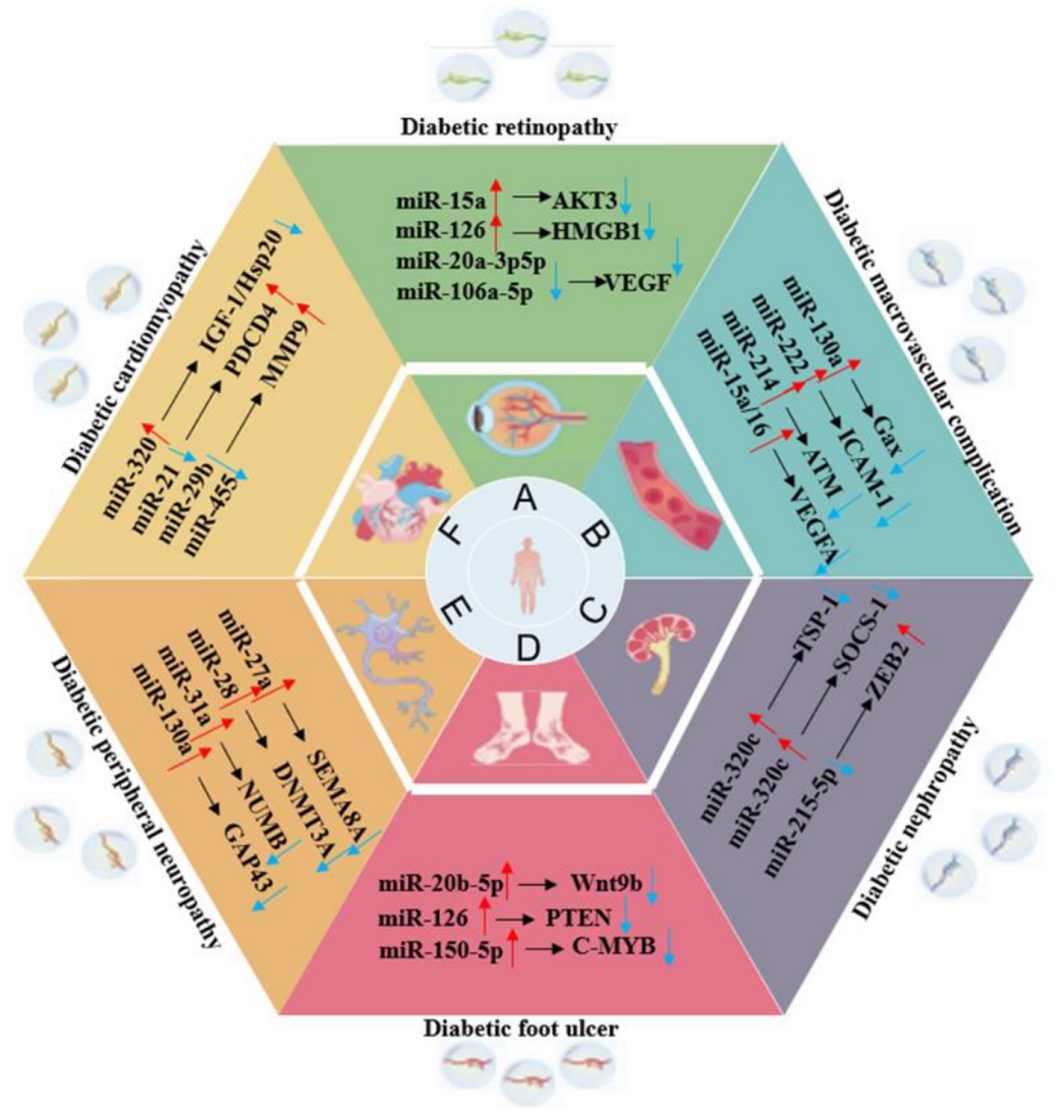
The role of exosomes in various organ complications of T2DM.

Exosomes, as a key medium for intercellular communication, have shown great potential in the treatment of multiple systemic complications of T2DM, as shown in Figure 4. These natural nanovesicles can precisely regulate multiple physiological system complications and organs across different tissues. Their unique natural targeting ability and low immunogenicity enable exosomes to act on multiple pathological processes simultaneously, breaking through the limitations of traditional single-target drugs. This treatment strategy, based on intercellular communication, offers a new idea for the collaborative management of T2DM complications and is expected to become an important breakthrough direction in the management of diabetes that involves multiple systems.

## 4 As the biomarker of disease

Exosomes are emerging as potential biomarkers because they reflect the physiological state of their originating cells, a characteristic that varies notably in metabolic diseases (95). This property highlights their clinical relevance for early diagnosis, disease monitoring, and evaluation of therapeutic outcomes. For example, the distinct expression of miRNAs in exosomes enables the early detection of diabetes (96), offering a more precise approach to disease management. Furthermore, regulating the production, release, and composition of exosomes presents promising avenues for therapeutic intervention in T2DM (97). A clinical trial demonstrated that miR-125a-5p exosomes derived from adipose tissue in the serum could serve as a causal biomarker for cognitive impairment in patients with T2DM (80). Such interventions hold the potential to provide personalized treatment plans tailored to the specific physiological and pathological profiles of patients.

As an emerging therapeutic approach and drug delivery system, exosomes offer significant advantages in treating diabetes and its complications. Compared to traditional treatments, such as insulin injections and oral hypoglycemic drugs, and artificial nano-drug carriers, exosomes exhibit higher biocompatibility, lower immunogenicity, and natural targeting properties, enabling them to effectively overcome biological barriers in drug delivery (80). Exosomes can also reverse immunosuppression under hyperglycemic conditions, not only delivering antibiotics for targeted sterilization but also reversing macrophage senescence and improving the microenvironment of diabetic wounds (54). Traditional antibiotic treatments often struggle to address both immunosuppression and bacterial resistance simultaneously. In contrast to synthetic nano-carriers like liposomes or polymer nanoparticles, exosomes offer superior tissue penetration and stability (98). The potential of exosomes in treating T2DM is attracting increasing attention. These small vesicles, which facilitate intercellular communication, can also function as biomarkers or drug-delivery systems.

Although exosome-based therapies for T2DM are still in the research phase, their future potential is significant. A deeper understanding of the biological properties of exosomes, their mechanisms, and their involvement in T2DM could lead to innovative therapeutic strategies in this field.

## 5 Limitations

Although exosome therapy shows great potential, there are still some challenges and limitations in successfully translating it into clinical practice. First, exosomes' size, contents, and functions vary greatly due to differences in cell source, cell state, and isolation methods. This high heterogeneity leads to poor reproducibility of research results and uncertainty in the stability and safety assessment of efficacy. Second, although exosomes have natural targeting properties, this targeting ability is often inefficient and non-specific. After systemic administration, the majority of exosomes are rapidly cleared by the mononuclear phagocytic system in the liver and spleen, and only a small portion can accumulate in the target tissues, which not only reduces efficacy but also may lead to off-target effects. Moreover, as a biologically active carrier, the contents of exosomes are complex and may contain oncogenic or proinflammatory molecules, posing potential safety risks. Although their immunogenicity is low, when heterologous exosomes are repeatedly injected, they may still trigger immune responses, leading to decreased efficacy or adverse reactions. These unknown safety variables are key issues that must be carefully evaluated in preclinical and future clinical studies. Third, exosomes exert their effects through a “multi-component-multi-target-multi-pathway” collaborative network, which is both an advantage and a barrier to defining them as a specific drug. Our current understanding of their exact mechanism of action, metabolic kinetics in the body, and optimal treatment dose and administration route is still very limited, making it extremely difficult to trace and control efficacy. In conclusion, the transition of exosome therapy from the laboratory to clinical practice still requires fundamental breakthroughs in production processes, safety assessment, and mechanism exploration.

## 6 Prospects

Exosome therapy still holds an extremely promising and exciting future, mainly manifested in the following aspects. First, engineered exosomes have the potential to achieve unprecedented, precise targeted treatment. By modifying their membrane surface, they can efficiently accumulate in specific tissues such as the damaged pancreas, kidneys, or retina. At the same time, by loading specific therapeutic molecules, they can precisely regulate key signaling pathways related to insulin resistance, cell apoptosis, or fibrosis, achieving “targeted” intervention at the root of the disease, thereby enhancing the therapeutic effect while minimizing systemic side effects. Second, exosomes' contents can serve as sensitive biomarkers for diagnosing T2DM and its different complications. In the future, the diagnostic markers of exosomes can be detected to assess the condition and be used as delivery carriers for therapeutic drugs to achieve dynamic monitoring and personalized treatment of the disease in a closed loop. Third, by using exosomes produced by the patient's own cells, highly individualized treatment plans can be developed, which effectively avoid immune rejection problems. The regenerative signals carried by these endogenous exosomes can not only protect and repair damaged β-cells and vascular endothelium, but also strongly promote the healing and regeneration of tissues.

## 7 Conclusions

This review systematically elaborates on how exosomes exert practical therapeutic effects in T2DM and its various complications through different key targets and signaling pathways. With their unique biological functions and engineering advantages, exosomes have opened up a highly promising new approach for the prevention and treatment of T2DM and its complications. Although there are still challenges in terms of standardization, targeting, and mechanism elucidation, with the deepening of research and the breakthrough of technical bottlenecks, it is expected to lead diabetes treatment from the traditional model to a new era of precise diagnosis and regenerative repair.
